# Temperature Sensitivity: A Potential Method for the Generation of Vaccines against the Avian Coronavirus Infectious Bronchitis Virus

**DOI:** 10.3390/v12070754

**Published:** 2020-07-14

**Authors:** Sarah Keep, Phoebe Stevenson-Leggett, Angela Steyn, Michael S. Oade, Isobel Webb, Jamie Stuart, Lonneke Vervelde, Paul Britton, Helena J. Maier, Erica Bickerton

**Affiliations:** 1 The Pirbright Institute, Pirbright, Surrey GU24 0NF, UK; sarah.keep@pirbright.ac.uk (S.K.); phoebe.stevenson-leggett@pirbright.ac.uk (P.S.-L.); angela.steyn@pirbright.ac.uk (A.S.); mso30@cam.ac.uk (M.S.O.); isobel.webb@pirbright.ac.uk (I.W.); jamiestuart20@gmail.com (J.S.); paul.britton@pirbright.ac.uk (P.B.); helena.maier@pirbright.ac.uk (H.J.M.); 2 Division of Infection and Immunity, The Roslin Institute and Royal (Dick), School of Veterinary Studies, University of Edinburgh, Easter Bush, Midlothian EH25 9RG, UK; lonneke.vervelde@roslin.ed.ac.uk

**Keywords:** coronavirus, IBV, temperature sensitivity, replicase, RNA synthesis

## Abstract

The *Gammacoronavirus* infectious bronchitis virus (IBV) is a highly contagious economically important respiratory pathogen of domestic fowl. Reverse genetics allows for the molecular study of pathogenic determinants to enable rational vaccine design. The recombinant IBV (rIBV) Beau-R, a molecular clone of the apathogenic Beaudette strain, has previously been investigated as a vaccine platform. To determine tissues in which Beau-R could effectively deliver antigenic genes, an in vivo study in chickens, the natural host, was used to compare the pattern of viral dissemination of Beau-R to the pathogenic strain M41-CK. Replication of Beau-R was found to be restricted to soft tissue within the beak, whereas M41-CK was detected in beak tissue, trachea and eyelid up to seven days post infection. In vitro assays further identified that, unlike M41-CK, Beau-R could not replicate at 41 °C, the core body temperature of a chicken, but is able to replicate a 37 °C, a temperature relatable to the very upper respiratory tract. Using a panel of rIBVs with defined mutations in the structural and accessory genes, viral replication at permissive and non-permissive temperatures was investigated, identifying that the Beau-R replicase gene was a determinant of temperature sensitivity and that sub-genomic mRNA synthesis had been affected. The identification of temperature sensitive allelic lesions within the Beau-R replicase gene opens up the possibility of using this method of attenuation in other IBV strains for future vaccine development as well as a method to investigate the functions of the IBV replicase proteins.

## 1. Introduction

Infectious bronchitis virus (IBV) is a *Gammacoronavirus* and is the aetiological agent of the acute highly contagious and economically important respiratory disease, infectious bronchitis, which affects domestic fowl. Other coronaviruses that cause respiratory disease are the zoonotic betacoronaviruses that can cause severe respiratory disease in humans including severe acute respiratory syndrome coronavirus (SARS-CoV) that emerged in 2003 [1,2], Middle East respiratory syndrome coronavirus (MERS-CoV) that emerged in 2012 [3] and SARS-CoV-2 that recently emerged in 2019 [4]. The alphacoronaviruses HCoV-229E, HCoV-NL63 as well as the betacoronaviruses HCoV-OC43 and HCoV-HKU1 also cause respiratory disease in humans but typically with less severe clinical symptoms [5]. The *Alphacoronavirus* porcine respiratory coronavirus (PRCV) is reported to inflict respiratory disease in pigs [6].

IBV is known to be predominantly spread through droplet and aerosol transmission [7]. There are several co-circulating strains of IBV, in terms of genotypes and serotypes, which inflict varying levels of disease, with vaccination against one often eliciting poor protective immune responses to another [8]. Infection with M41, a Massachusetts serotype and one that is of particular relevance to the global poultry industry [8], results in classical IB symptoms including snicking, rales, watery eyes, nasal discharge, lethargy, reduced weight gain in broilers and reduced egg laying as well as egg quality in layers. In addition, nephropathogenic strains of IBV have been identified in which viral replication is not confined to the respiratory tract with replication occurring in the kidneys. Nephropathogenic IBV strains, such as QX and B1648, are able to disseminate, initially from the respiratory tract, through the bird and infect the enteric tract, oviducts and kidneys, often resulting in severe renal disease [9,10,11].

Vaccination against IBV is commonly practiced using live attenuated vaccines administered to young chicks en masse through sprays or via drinking water, with older layer birds typically boosted with inactivated vaccines [8]. All current live attenuated vaccines are produced through serial passage of a virulent field isolate through embryonated hens’ eggs, typically 80 to 100 times [12,13]. A fine balance between attenuation and the retention of immunogenicity must be achieved and although this process of vaccine development is well established, the molecular mechanisms are poorly understood. Recent research has indicated only a few consensus level mutations are acquired over the passaging process presenting a short possible route back to virulence [14]. There is therefore a drive to understand pathogenic determinants present in the IBV genome, to enable the generation of rationally attenuated IBV vaccines.

The advent of reverse genetics systems has opened the potential for developing rationally attenuated IBV viruses as vaccines [15,16,17,18]. The recombinant IBV (rIBV) Beau-R, a molecular clone of the attenuated Beaudette strain Beau-CK [19], has been investigated as a vaccine backbone. Several studies have demonstrated that Beau-R expressing an S gene from a pathogenic isolate, can induce a protective immune response against the pathogenic wild type IBV strain from which the “donor” S sequence was derived [15,16,20]. The vaccine strain H52 lacking accessory genes 3 and 5 can induce a protective immune response against homologous challenge [17]. A recombinant H120 expressing the S1 subunit from a QX like strain induced a better protection rate to homologous challenge in comparison to vaccination with H120 [18]. Whilst the research is promising, the ultimate question of how these vaccine virus backbones are attenuated is still unanswered. The H52 and H120 backbones were generated through serial passage in embryonated hens’ eggs [12] and the complete passage history of the Beaudette strain, first isolated in 1937, is unknown although reports suggest this strain has been passaged upward of 200 times [15]. The Beaudette strain is a particular enigma as it is thought that the virus is so attenuated it can no longer initiate effective replication in vivo nor induce any protective immune response [15,21].

In this study the in vivo dissemination of the attenuated rIBV Beau-R was compared to the pathogenic strain M41-CK. Beau-R replication was found to be highly restricted with infectious virus recovered from the beak but not from the trachea. Replication of IBV in tracheal epithelial cells has long been associated with clinical signs and pathology associated with IB in chickens. In vitro assays identified that Beau-R replication is temperature sensitive and cannot be maintained at 41 °C, the core body temperature of a chicken. Utilising a panel of rIBV, the replicase gene was identified as a determining factor in the restriction of the replication of Beau-R at the non-permissive temperature. The inability of Beau-R to replicate at 41 °C likely contributes to the attenuated in vivo phenotype associated with IBV Beaudette. Whilst this finding has implications for the use of Beau-R as a backbone for the development of rationally designed recombinant vaccines, it supplements previous research that the coronavirus replicase gene is a determinant of pathogenicity [22,23,24,25,26,27,28]. It also highlights the possibility, with greater understanding, that temperature sensitivity could be used as a method for the design of rationally attenuated vaccine viruses as currently seen with viral pathogens including influenza virus and rhinoviruses [29,30,31,32].

## 2. Materials and Methods

### 2.1. Cells and Viruses

Primary chicken kidney (CK) cells were prepared from kidneys extracted from two three-week-old specific pathogen free (SPF) Rhode Island Red (RIR) chickens [33]. DF1 cells are a continuous cell line derived from chicken embryo fibroblasts [34]. All cell cultures were produced by the Central Services Unit at The Pirbright Institute and maintained, prior to infection, at 37 °C and 5% CO_2_.

All viruses were propagated in embryonated RIR hens’ eggs, supplied by the National Avian Facility (NARF), The Roslin Institute. Allantoic fluid was clarified by low speed centrifugation and either titrated in CK cells in triplicate or in ex vivo tracheal organ cultures (TOCs) as described previously [15]. Titres are displayed as either the number of plaque forming units (PFU) per ml or as the dose required to causes 50% ciliostasis (CD50). The rIBVs Beau-R [19], BeauR-M41(S) [35] and BeauR-M41-Struct [23] have been described previously, as have the laboratory strains M41-CK [35], Beau-CK [19] and serially egg passaged viruses M41-CK EP106 A, A1, C and D [14]. The H120 vaccine strain was a gift from Professor R. Jones at the University of Liverpool and nephropathogenic strain D388 [36], a gift from Professor J.J de Wit at The GD Animal Health, Netherlands.

### 2.2. In Vivo Characterisation

All animal experimental protocols were carried out in strict accordance with the UK Home Office guidelines and under licence granted for experiments involving regulated procedures on animals protected under the UK Animals (Scientific Procedures) Act 1986. The experiment was performed in The Pirbright Institute Home Office licenced (X24684464) experimental animal house facilities and were approved (AR000772, 4 August 2016) by the animal welfare and ethical review committee under the terms of reference HO-ERP-01-1.

SPF RIR chickens were housed in raised floor pens in separate positive pressure high-efficiency particulate air (HEPA) filtered rooms in groups of 24 birds. Chickens were inoculated with 100 µL containing 10^4^ PFU of rIBV Beau-R or IBV M41-CK or 100 µL Phosphate Buffered Saline (PBS) for mock infection via the intra-nasal and intra-ocular route at seven days of age. Clinical signs, including rales and snicking were observed from one to seven days post infection (dpi) as previously described [20]. One, four and six dpi, six randomly selected birds per group were culled by cervical dislocation. A variety of tissues were harvested with sections stored in either PBS or RNALater (Ambion, Thermo Fisher Scientific, Waltham, MA, USA). On day seven, post infection, all remaining birds were euthanized by cervical dislocation. Ciliary activity was assessed in trachea extracted 4 and 6 dpi as previously described [37,38].

### 2.3. Virus Re-Isolation in Embryonated Hens’ Eggs

Infectious virus was re-isolated from harvested tissues in ten-day old SPF Valo embryonated hens’ eggs. Tissue was homogenised using a tissue lyser II (Qiagen, Hilden, Germany), in 500 µl PBS containing 10 U/mL penicillin, 10 µg/mL streptomycin and 50 U/mL nystatin followed by clarification by low speed centrifugation. Each egg was inoculated with 100 µL clarified tissue derived supernatant and incubated for 24 h at 37 °C. Embryos were culled using a schedule 1 method and the allantoic fluid harvested, which was screened for viral presence by RT-PCR utilising primers that target the 3′ UTR as described previously [23].

### 2.4. In Vitro Growth Kinetic Experiments

DF1 or CK cells, seeded in six or twelve well plates, were infected with 10^4^ PFU, 10^5^ PFU, or 1.5 log_10_CD50 (MOI ~0.001–~0.01) rIBV or IBV, and incubated for 1 h at 37 °C, 5% CO_2_ or 41 °C, 5% CO_2_. Inoculum was removed, and cells were washed twice with PBSa to remove unbound virus. Per well, 3 mL BES (N,N-Bis(2-hydroxyethyl)-2aminoethanesulphonic acid) medium [39] was added, and cells were incubated at either 37 °C, 5% CO_2_ or 41 °C, 5% CO_2_. Supernatant containing viral progeny, one well per time point, was harvested at defined intervals post infection (pi) and titrated in triplicate in CK cells. Each experiment was performed three times.

### 2.5. Analysis of sgmRNA Synthesis by Quantitative Real-Time PCR (qRT-PCR)

CK cells seeded in 6 well plates were inoculated with 10^6^ PFU (multiplicity of infection (MOI) ~0.01) of Beau-R or M41-CK and incubated at either 37 °C or 41 °C. After 24 hpi, supernatant was harvested and assessed for infectious progeny by titration in CK cells. Cell lysate was also harvested, and RNA was extracted using RNeasy columns (QIAgen), following the manufacturer’s instructions, including on column DNAse treatment. Reverse transcription of 1 μg total RNA was performed using the Superscript III Reverse Transcriptase kit (Invitrogen, Waltham, MA, USA) according to the manufacturer’s protocol using random primers (Promega, Madison, WI, USA). The viral RNA load was quantified using quantitative real-time PCR (qRT-PCR) using primers targeting the N sub-genomic mRNA (sgmRNA). Quantitative RT-PCR was performed using TaqMan Universal Master Mix II, no UNG (Life Technologies, Waltham, MA, USA) including 125 nM final probe and 500 nM final primers. The primer and probe sequences were taken from Maier et al. (2013) [40] and are as follows: forward primer located in the leader sequence, 5′-CTAGCCTTGCGCTAGATTTTTAACT-3′; reverse primer located in the N gene 5′-GAGAGGTACACGCGGGACAA-3′; and N sgmRNA probe sequence, 5′-FAM-ACAAAGCAGGACAAGCA-MGB-NFQ-3′. Standard curves were performed to allow quantitation of IBV RNA copy numbers based on cDNA levels using plasmids containing the sequence amplified by the set of primers listed above. The resulting Ct results were used to calculate the log of relative RNA copies (Log10) using the linear equation from the standard curve.

### 2.6. Temperature Swap Assays

CK cells seeded in six well plates were inoculated with 10^5^ PFU (MOI ~0.01) of rIBV or IBV. Infected cells were incubated at either 37 °C or 41 °C for 1 h (1st incubation), after which the inoculum was removed, and cells washed with PBSa to remove unbound virus. Per well, 3 mL BES media was added, and cells were incubated for a further 23 h at either 37 °C or 41 °C (2nd incubation). Supernatant was harvested from three wells for each experimental condition, and each was titrated in triplicate in CK cells to determine the quantity of infectious progeny virus. Each experiment was performed three times.

### 2.7. Co-Infection Assay

CK cells seeded in six well plates were inoculated in triplicate with 10^5^ PFU (MOI ~0.01) of M41-CK, Beau-R, co-infected with 10^5^ PFU Beau-R and 10^5^ PFU M41-CK or mock infected with BES media. Cells were incubated at 37 °C, 5% CO_2_ or 41 °C, 5% CO_2_ for 1 h, after which the inoculum was removed, and cells washed once with PBSa to remove any unbound virions. Per well, 3 mL BES media was added, and cells were incubated at either 37 °C or 41 °C until extensive CPE was observed in the 37 °C Beau-R and M41-CK control samples, typically 48 to 72 hpi. The supernatants were harvested and passaged a further four times in CK cells, using the same incubation conditions as the original passage. Passage 5 supernatant was screened for viral presence using RT-PCR utilising primers targeting the 3′ UTR, described previously [23].

### 2.8. Analysis of Protein Expression by Western Blot

CK cells were seeded in six well plates and inoculated with 10^6^ PFU M41-CK, Beau-R or BES media for mock infection and incubated at either 37 °C or 41 °C. At 8, 11 and 24 hpi cell lysates were harvested using radioimmunoprecipitation assay (RIPA) lysis buffer (Thermo Fisher Scientific, Waltham, MA, USA) with Protein Inhibitor Cocktail added (Thermo Fisher Scientific). Cell lysates were resolved using sodium dodecyl sulphate-polyacrylamide gel electrophoresis (SDS-PAGE) and were then transferred to a nitrocellulose membrane. Non-specific binding sites were blocked using a 5% milk solution in PBS-tween (PBS-T) for 1 h at room temperature. The blots were probed with anti-IBV primary antibody (dilution 1:1000, Abcam, ab31671) and β-actin (dilution 1:2000, Abcam, ab8227). Bound antibodies were detected with Donkey anti-Chicken (dilution 1:15,000, Licor, 926-68075) and Goat anti-Rabbit (dilution 1:15,000, Abcam, ab150077). Visualisation of membrane was carried out using the LICOR Odyssey CLx Imaging System.

### 2.9. Statistics

Each figure details the statistical tests used to analyse the data set presented. Graphpad Prism, version 7.0 was used for all statistical analysis. Prior to each statistical test the normality of each data set was assessed.

## 3. Results

### 3.1. Replication of rIBV Beau-R is Restricted In Vivo

**Figure 1:**
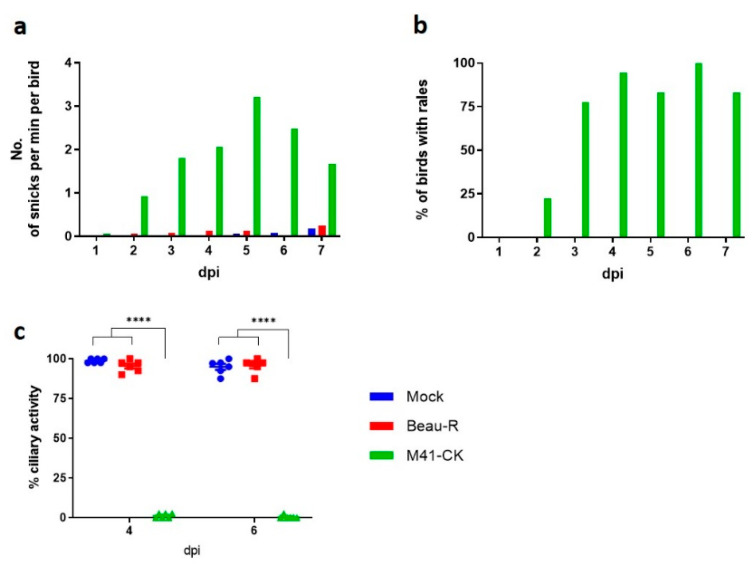
Beau-R displays an attenuated phenotype in vivo. SPF chickens were inoculated at 7 days of age with 10^4^ PFU of infectious bronchitis virus (IBV) strain Beau-R, M41-CK or PBS for mock infection. The birds were assessed from 2 to 7 dpi for the presence of clinical signs. (**a**) The average number of snicks per bird was calculated and (**b**) the percentage of birds exhibiting rales. Only birds infected with M41-CK displayed clinical signs. (**c**) Ciliary activity was measured in trachea harvested from six randomly selected birds (*n* = 6) at 4 and 6 dpi. The average percentage for each group is displayed with error bars representing standard error of the mean (SEM). Statistical differences were assessed using a two-way ANOVA followed by Tukey analysis for multiple comparisons and are represented by **** (*p* < 0.0001).

In order to establish the in vivo sites of Beau-R replication, groups of seven-day old SPF RIR chickens were inoculated with either M41-CK, Beau-R or mock infected with PBS. Clinical signs including snicking (Figure 1a) and rales (Figure 1b) were assessed from 1–7 dpi. No IBV induced clinical signs were observed in the mock group or the birds infected with Beau-R. In contrast, chickens infected with M41-CK displayed snicking and rales from 2 dpi, with the rate of snicking peaking at 5 dpi and the percentage of birds with rales peaking at 6 dpi. Tracheas were harvested from six randomly selected birds per group, 4 and 6 dpi and the ciliary activities assessed. Ciliary activities of tracheal epithelial cells are used as a marker to determine whether a pathogenic isolate of IBV is present [41]. The mean ciliary activities were comparable between the mock and Beau-R infected birds, 99% and 95% respectively, 4 dpi and 95% and 96%, respectively, 6 dpi. In contrast, M41-CK infected birds exhibited ciliary activities of 1% and 0.4% (*p* < 0.0001) on days 4 and 6 dpi, respectively (Figure 1c). The absence of clinical signs together with the retention of ciliary activities confirms previous the reports that Beau-R has an attenuated phenotype in vivo [15,16,42].

To determine the sites of viral replication for Beau-R and M41, eyelids, beak and tracheas were harvested from six randomly selected chickens per group at 1, 4, 6 and 7 dpi. For the purpose of this study, the beak refers to all soft tissue within the beak cavity including the nasal turbinates and nasal-associated lymphoid tissue (NALT), and eyelids includes both the upper and lower lid. To determine whether infectious progeny virions were being produced in the selected tissues, embryonated hens’ eggs were inoculated with tissue-derived supernatants and the resulting allantoic fluid analysed by RT-PCR (Table 1) for the presence of IBV RNA using a primer set specific for the 3′ UTR [23]. Due to a 184 bp deletion within the 3′ UTR of the M41-CK genome, the RT-PCR products generated from this primer set can be easily distinguished, on size difference, between M41-CK and Beau-R derived RNA [23]. As expected, no infectious Beau-R was isolated from tracheal samples on any sampling day, compared to at least 50% of tracheal samples testing positive for the presence of M41-CK on each sampling day post-infection. M41-CK was detected in eyelid tissue harvested from all birds 4 and 6 dpi and in four of six birds and five of six birds, 1 and 7 dpi respectively. Beau-R was not detected in the harvested eyelid tissue except for one bird at day 7 dpi. All beak tissue, however, tested positive for the presence of Beau-R at 1 dpi, which was comparable to M41-CK at this time point, but the detection of Beau-R dropped to ≤50% at 4 and 7 dpi with none detected at 6 dpi. These results indicate that Beau-R replication is confined to tissue within the beak, and largely restricted to early infection.

### 3.2. Replication of rIBV Beau-R In Vitro Is Sensitive to Temperature

The observation that in vivo replication of Beau-R is essentially restricted to the tissue within the beak raised an interesting question regarding the potential for temperature to affect replication and therefore viral dissemination through the host. The continuous movement of air in the upper respiratory tract will inevitably cool this area of the bird, and it has been demonstrated for several human respiratory viruses including influenza virus [43,44], Rhinovirus [30,31] and respiratory syncytial virus (RSV) [45,46,47] that replication can be confined to the upper respiratory tract as a consequence of viral replication being sensitive to the comparatively higher temperatures in the lower respiratory tract. A growth kinetics assay in CK cells was used to assess whether the replication of Beau-R was detrimentally affected by an increase in temperature. The core body temperature of a chicken is considered to be 41 °C [48], and therefore this temperature was chosen for all experiments comparing productive replication to standard conditions using an incubation temperature of 37 °C.

**Figure 2:**
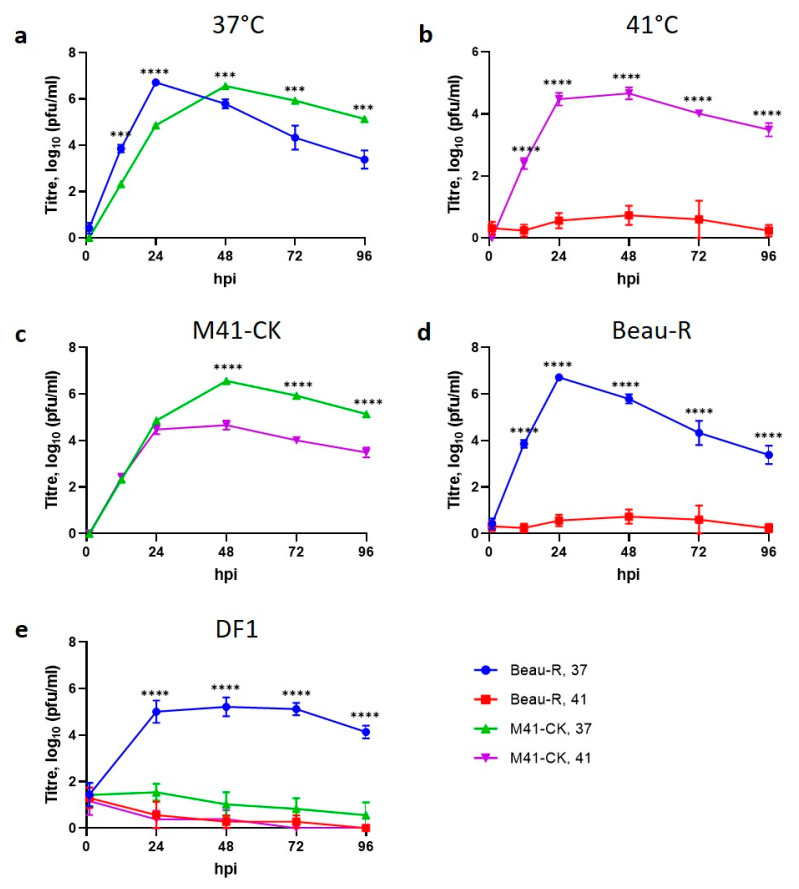
The in vitro replication of Beau-R is sensitive to temperature. Primary chicken kidney (CK) cells seeded in 6 well plates were inoculated with 10^4^ PFU (MOI ~0.001) of Beau-R or M41-CK and incubated at either 41 °C or 37 °C. Supernatants were harvested at 24 h intervals and the quantity of infectious progeny determined via titration in CK cells. (**a**) Represents the data generated through incubation at 37 °C, (**b**) incubation at 41 °C, (**c**) data relating to M41-CK infection and (**d**) Beau-R infection. (**e**) The same assay utilising 10^5^ PFU (MOI ~0.01) of Beau-R and M41-CK was carried out in DF1 cells. Each point represents the mean of three independent experiments (*n* = 3) with error bars representing SEM. Statistical differences were assessed using a two-way ANOVA followed by a Tukey test for multiple comparisons and are highlighted by *** (*p* < 0.0005) and **** (*p* < 0.0001).

Primary CK cells were inoculated with Beau-R or M41-CK and incubated at either 41 °C or 37 °C, with supernatant harvested at regular intervals (Figure 2a–d). The titres of infectious Beau-R determined from cells incubated at 41 °C were lower than those from cells incubated at 37 °C (*p* < 0.0001), with very little viable infectious progeny produced at the higher temperature (Figure 2b). In contrast, the quantity of infectious progeny released from cells infected with M41-CK was unaffected by temperature at 12 and 24 hpi (Figure 2c). The titres of M41-CK determined from CK cells incubated at 41 °C were observed to be lower at 48 to 96 h (*p* < 0.0001) than those determined, at similar timepoints, from cells at 37 °C. Although it is clear, from these results, that the replication of both M41-CK and Beau-R is negatively affected at 41 °C, there is a substantial observable difference in the extent to which each virus is affected. M41-CK is able to sustain a level of replication capable of generating consistent titres around 104 PFU/mL, whereas Beau-R was unable to sustain any productive infection at the higher temperature (Figure 2c,d). These results demonstrate that a temperature of 41 °C can be considered as a non-permissive temperature for Beau-R replication.

To ensure that the temperature sensitive replication phenotype observed for Beau-R was not specific to the primary CK cells, the replication of both M41-CK and Beau-R was assessed in DF1 cells (Figure 2e), a continuous cell line derived from chicken embryo fibroblasts [34]. Similar to the results observed in CK cells, Beau-R replication was negatively affected by incubation at 41 °C (*p* < 0.0001, Figure 2d), indicating the temperature sensitive replication phenotype for Beau-R was not specific to cell type. The titres of M41-CK produced at either temperature were below 10 PFU/mL demonstrating that M41-CK could not initiate productive replication in DF1 cells. This was not unexpected as the M41-CK spike (S) glycoprotein restricts cell tropism [35]. Whilst it may seem counterintuitive to include M41-CK in the assay, it was not known whether temperature affected S mediated entry, allowing viruses to enter cells previously non-permissive to infection.

### 3.3. Temperature Sensitivity Is Not a Shared Characteristic of Attenuated Strains

The observation that replication of the attenuated strain Beau-R was highly restricted at 41 °C whereas the pathogenic M41-CK strain could both initiate and sustain replication raised the possibility that temperature sensitivity may be an explanation for attenuation of IBV in vivo. To investigate this possibility, the replication kinetics at 37 °C and 41 °C in CK cells of the IBV vaccine strain H120 were assessed, as were the replication kinetics of the serially passaged M41-CK isolates, M41-CK EP106 A, M41-CK EP106 A1, M41-CK EP106 C and M41-CK EP106 D [14]. Notably, all these viruses have been attenuated through serial passage in embryonated hens’ eggs (120 and 106 passages respectively) [12,14], a process that typically occurs at 37 °C. It was hypothesized, therefore, that this process may have resulted in attenuation of the viruses through selection of a temperature sensitive phenotype causing a restriction in replication at a higher temperature.

**Figure 3:**
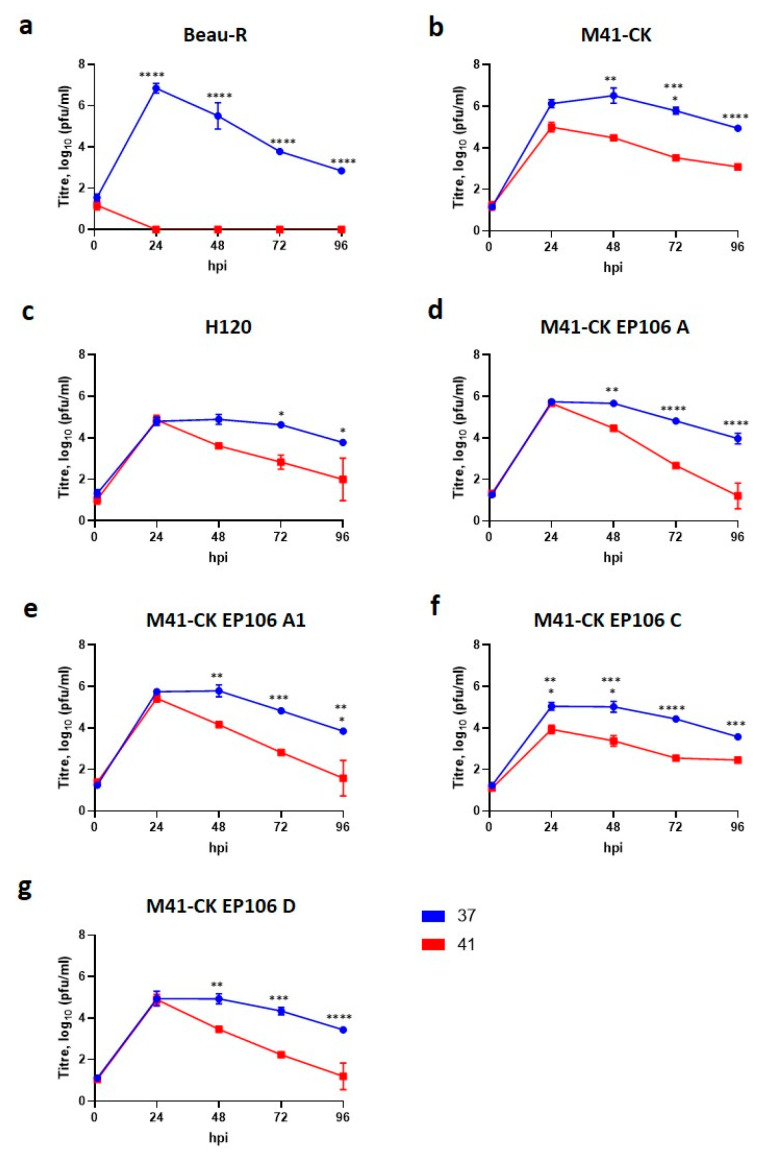
The attenuated strains H120 and M41-CK EP106 A–D do not share a temperature sensitive replication phenotype comparable to Beau-R. Primary CK cells seeded in 6 well plates were inoculated with 10^5^ PFU (MOI ~0.01) of (**a**) Beau-R, (**b**) M41-CK, (**c**) H120, (**d**) M41-CK EP106 A, (**e**) M41-CK EP106 A1, (**f**) M41-CK EP106 C or (**g**) M41-CK EP106 D, and incubated at either 37 °C or 41 °C. Supernatants were harvested at 24 h intervals and the quantity of infectious progeny determined via plaque assay in CK cells. Each point represents the mean of three independent experiments (*n* = 3) with error bars representing SEM. Statistical differences were assessed using a two-way ANOVA followed by a Tukey test for multiple comparisons and are highlighted by * (*p* < 0.05), ** (*p* < 0.005), *** (*p* < 0.0005) and **** (*p* < 0.0001).

All of the attenuated IBVs investigated were able to initiate and sustain productive replication at 41 °C, similar to the phenotype observed for pathogenic M41-CK (Figure 3a–g). The titres produced from H120 infected cells at 24 hpi were comparable between those cells incubated at 37 °C and those at 41 °C (Figure 3c). This was also the case for isolates M41-CK EP106 A, M41-CK EP106 A1 and M41-CK EP106 D (Figure 3d–g). Interestingly, all the viruses investigated exhibited decreased titres of infectious progeny at 41 °C, in comparison to 37 °C, at 48 to 96 hpi (*p* < 0.005), as previously observed for M41-CK. None of the viruses exhibited the temperature sensitive replication phenotype observed for Beau-R (Figure 3a). These results showed that attenuation associated with H120 and the multiple passaged isolates of M41-CK viruses were not due to a restriction in replication linked to temperature sensitivity.

### 3.4. The Nephropathogenic IBV Strain D388(QX) Is Able to Replicate at 41 °C

Although the results described above and in Figure 3 show that temperature sensitivity is not a shared characteristic of attenuated strains, it also possible that the ability to replicate at 41 °C is not necessarily a trait shared by other pathogenic strains. The observation from our in vivo results that Beau-R was not detected in tracheal epithelial cells, raised the question of whether attenuation and resultant viral dissemination was linked to restricted replication resulting from temperature sensitivity at the core temperature (41 °C) of a chicken. It is therefore important to determine whether the ability of pathogenic M41-CK to grow at 41 °C is a trait shared by other pathogenic strains, particularly with regards to nephropathogenic strains of IBV that disseminate through the bird and infect the kidneys.

**Figure 4:**
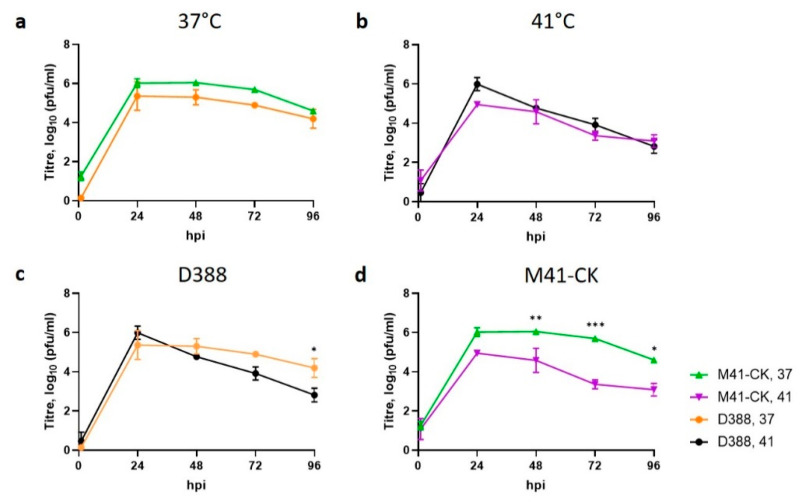
The replication of D388(QX) is unaffected by temperature. Primary CK cells seeded in 12 well plates were inoculated with 1.5 log10CD50 (MOI ~ 0.001) of M41-CK or D388(QX) and incubated at 41 °C or 37 °C. Supernatants were harvested at 24 h intervals and the quantity of infectious progeny determined via plaque assay. Graph (**a**) represents data generated at 37 °C and (**b**) at 41 °C. Graph (**c**) represents all data relating to D388(QX) infection and (**d**) M41-CK. Each point represents the mean of three independent experiments (*n* = 3) with error bars representing SEM. Statistical differences were assessed using a two-way ANOVA followed by a Tukey test for multiple comparisons and are highlighted by * (*p* < 0.05), ** (*p* < 0.005) and *** (*p* < 0.0005).

The replication kinetics of the nephropathogenic strain D388(QX), a strain associated with severe infectious bronchitis, resulting from infection in the respiratory tract and also with dissemination to and resultant pathology in the kidneys and oviducts [36], were assessed at 37 °C and 41 °C and compared to M41-CK (Figure 4). Overall, replication of D388(QX) was found to be largely unaffected by temperature with titres comparable between the two temperatures at 24 and 48 hpi (Figure 4c). Titres of infectious progeny were lower at 72 and 96 hpi from cells incubated at 41 °C, however statistical significance was only reached for the later time point (*p* < 0.05). The ability of D388(QX) to establish and maintain replication at 41 °C, like M41-CK, suggests that whilst attenuation and temperature sensitivity may not be intrinsically linked, the ability to replicate at higher temperatures may be a shared characteristic of pathogenic IBV isolates.

### 3.5. Entry at Permissive Temperatures Cannot Recover Replication at Non-Permissive Temperatures

**Figure 5:**
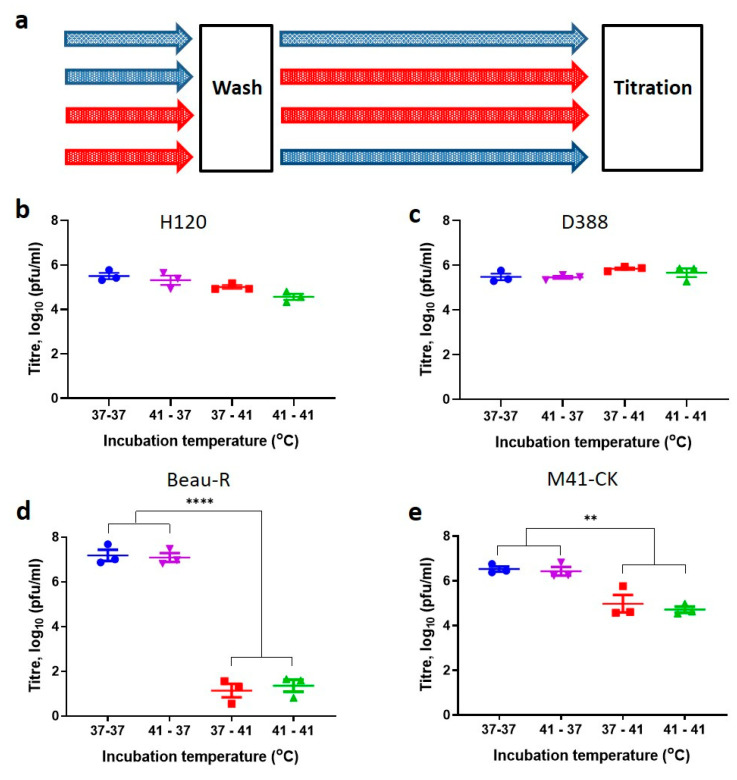
Entry is not the determining factor to whether productive replication is initiated at 41 °C**.** (**a**) Schematic detailing experimental protocol. Primary CK cells seeded in 6 well plates were inoculated in triplicate with 10^5^ PFU of IBV (MOI ~0.01). Cells were incubated for 1 h at either 37 °C (blue) or 41 °C (red), after which the inoculum was removed and the cells washed to remove unbound virus. Cells were incubated at either 37 °C or 41 °C for 23 h. The quantity of infectious viral progeny produced was assessed through titration in CK cells. Graph (**b**) represents data from H120, (**c**) D388(QX), (**d**) Beau-R and (**e**) M41-CK infection. Each point represents the mean from three independent experiments (*n* = 3) with error bars indicating SEM. Statistical differences were assessed using a one-way ANOVA with a Tukey test for multiple comparisons and are highlighted by ** (*p* < 0.005) and **** (*p* < 0.0001).

In order to elucidate which step in the IBV replication cycle accounted for the temperature sensitive replication phenotypes observed, a temperature swap experiment was carried out (Figure 5a). CK cells were inoculated and incubated at either 37 °C or 41 °C for 1 h to allow for viral attachment and entry. The inoculum was removed, and the cells washed to remove unbound virus, the cells were then either incubated at the same temperature or incubated at the alternative temperature for a further 23 h (Figure 5a). The resulting supernatants were assessed for infectious viral progeny through titration in CK cells at 37 °C.

The titres of infectious progeny from CK cells infected with H120 and D388(QX) were unaffected by a swap in temperature (Figure 5b,c); this was expected as previous results demonstrated that replication is not affected by temperature at 24 hpi (Figure 3c and Figure 4). The titres of both Beau-R and M41-CK were affected by the swaps in temperature (Figure 5d,e), with Beau-R affected to a greater extent than M41-CK. The titres of viral progeny produced were comparable in cells incubated at 37 °C for binding and entry and then 37 °C for the remainder of the replication cycle (37–37) and those incubated at 41 °C and then 37 °C (41–37), indicating that a difference in the binding and entry step temperature has no effect on the viral replication step. This indicates that the temperature in which viral attachment and entry occurred was not the determining factor in establishing a productive infection. In contrast, titres were significantly lower when cells were incubated at 41–41 in comparison to 41–37, and also between 37–37 and 37–41 (*p* < 0.005), demonstrating that a change in the second incubation temperature affected viral replication. This therefore indicates that the temperature at which the virus is undergoing active replication, assembly and egress was the determining factor in establishing a productive infection.

### 3.6. The Spike Glycoprotein from M41-CK Cannot Rescue Beau-R Replication at 41 °C

**Figure 6:**
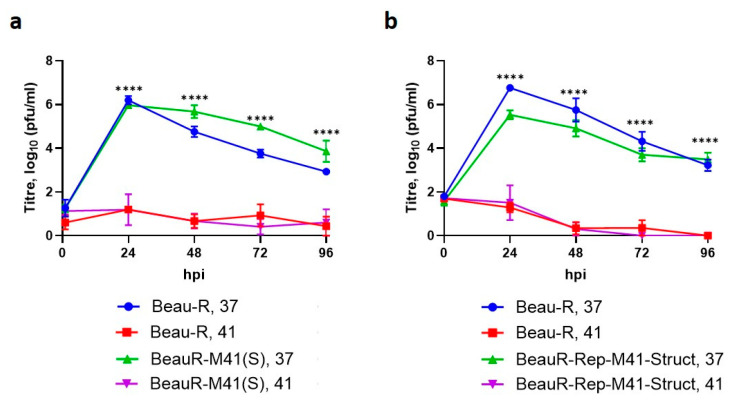
The structural and accessory genes from M41-CK cannot rescue Beau-R replication at 41 °C. Primary CK cells seeded in 6 well plates were inoculated with (**a**) 10^4^ PFU (MOI ~0.001) of Beau-R or BeauR-M41(S) and (**b**) 10^5^ PFU (MOI ~0.01) of either Beau-R or BeauR-M41-Struct and incubated at either 37 °C or 41 °C. Supernatants were harvested at 24 h intervals the quantity of infectious progeny determined via titration in CK cells. Each point represents the mean of three independent experiments (*n* = 3) with error bars representing SEM. Statistical differences were assessed using a two-way ANOVA followed by a Tukey test for multiple comparisons. Differences are highlighted by **** (*p* < 0.0001) (**a**) between Beau-R at 37 °C and Beau-R 41 °C, as well as BeauR-M41(S) at 37 °C and 41 °C and (**b**) between Beau-R at 37 °C and Beau-R at 41 °C as well as BeauR-M41-Struct at 37 °C and BeauR-M41-Struct at 41 °C.

Coronavirus entry is mediated by the S glycoprotein [49]. In order to exclude viral entry as the determining factor for the replication of Beau-R at non-permissive temperatures, a rIBV, BeauR-M41(S), in which the Beaudette S gene was replaced with corresponding gene from M41-CK, was investigated [35]. Analysis of the growth kinetics at the two temperatures, 37 °C and 41 °C, revealed that BeauR-M41(S) displayed comparable growth kinetics to Beau-R at 41 °C (Figure 6a). This result demonstrated that the M41 S glycoprotein could not confer the ability of M41-CK to replicate at 41 °C thus confirming that the IBV receptor binding protein is not responsible for the restricted growth of Beau-R at 41 °C.

### 3.7. The Replicase Gene Is a Determinant of Temperature Sensitivity

The results of the temperature swap assays, alongside the inability of BeauR-M41(S) to replicate at 41 °C, indicated that it was not the temperature at which viral entry occurred, but rather the temperature at which active viral replication took place which determined whether a productive infection was established. In order to rule out a role for another structural or accessory protein in the restricted temperature phenotype for Beau-R, a second rIBV, BeauR-M41-Struct [23], was investigated. BeauR-M41-Struct contains Beau-R derived sequence encoding the 5′ UTR and replicase gene, with the structural and accessory genes plus the 3′ UTR sequences from M41-CK. Analysis of the growth kinetics of BeauR-M41-Struct at the two temperatures revealed that this rIBV, like Beau-R and BeauR-M41(S), could not establish productive infection at 41 °C (Figure 6b). This result demonstrated that the M41-CK derived structural and accessory genes could not confer the ability of M41-CK to replicate at 41 °C. This in turn, indicates that the replicase gene is the determining factor in the temperature sensitivity associated with the replication of Beau-R.

### 3.8. Temperature Affects sgRNA Levels during Beau-R Infection

**Figure 7:**
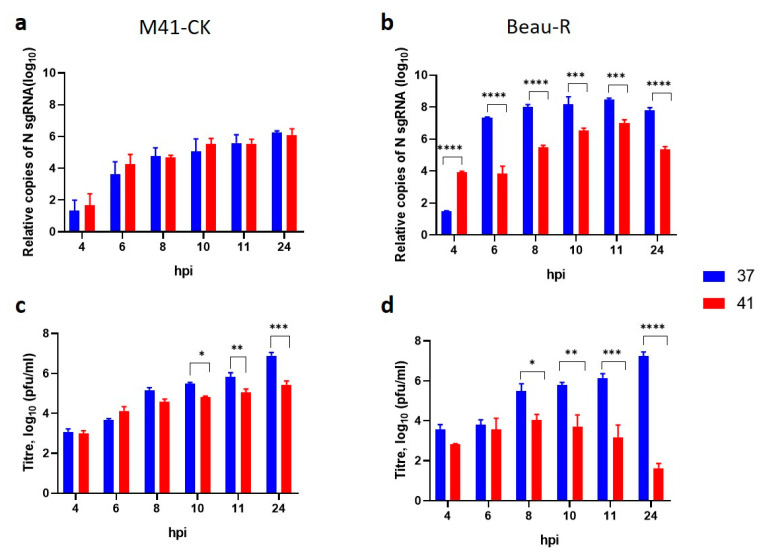
Temperature affects the level of sgRNA during Beau-R infection. Primary CK cells seeded in 6 well plates were inoculated with 10^6^ PFU (MOI ~0.1) of M41-CK (**a**,**c**) or Beau-R (**b**,**d**) and incubated at either 37 °C or 41 °C. Cell lysates and supernatants were harvested at regular intervals. (**a**,**b**) RNA was extracted from the cell lysates and assessed by RT-qPCR for the quantity of the IBV N protein sgRNA as described by Maier et al., (2013). (**c**,**d**) The quantity of infectious progeny in the supernatant was assessed via titration in CK cells. In all graphs, the mean of three independent experiments (*n* = 3) is presented and error bars represent SEM. Statistical differences were assessed using a two-way ANOVA with Sidak analysis for multiple comparisons and are highlight by * (*p* < 0.05), ** (*p* < 0.005), *** (*p* < 0.0005) and **** (*p* < 0.0001).

As the IBV replicase gene had been identified as a factor in temperature sensitivity, it was hypothesised that increasing temperature was negatively affecting the level of RNA synthesis, causing a reduction in the number of infectious progeny. The coronavirus replicase is responsible both for de novo synthesis of the full-length genomic RNA thus generating genomic RNA for progeny viruses as well as generating the replicase proteins, and for the synthesis of a set of subgenomic RNAs (sgRNA), that are used for the generation of the structural and accessory proteins. In order to investigate whether the restriction in growth observed for Beau-R at 41 °C was linked to RNA expression a series of infections were used to investigate whether the quantities of sgRNA synthesised differed at the two different temperatures. Specifically, the levels of the sgRNA responsible for the expression of nucleocapsid (N) protein was used as an indicator for the level of viral RNA synthesis (Figure 7). The quantities of infectious viral progeny were determined for a comparison between viral RNA synthesis and productive virus replication.

**Figure 8:**
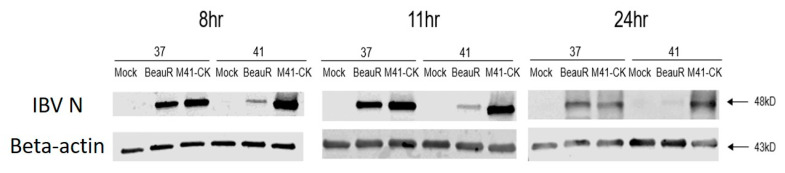
During Beau-R infection the quantity of N protein is less at 41 °C than at 37 °C. Primary CK cells seeded in 6 well plates were inoculated with 10^6^ PFU (MOI ~0.1) Beau-R or M41-CK or N,N-Bis(2-hydroxyethyl)-2aminoethanesulphonic acid (BES) media for mock infection, and incubated at either 37 °C or 41 °C. Cell lysates were harvested at 8, 11, and 24 hpi. Proteins present in the cell lysates were denatured using SDS lysis buffer and separated on a 4–15% polyacrylamide gel. The amounts of the IBV N protein present in the samples were analysed by western blot using an anti-IBV antibody. Levels of actin were analysed using an anti-β-actin as a loading control. Identified bands relate to β Actin (43kDa) and the IBV N protein (48kDa).

At all the time points assessed, M41-CK N sgRNA levels were largely comparable in cells incubated at 37 °C and those incubated at 41 °C (Figure 7a), indicating that sgRNA synthesis is not affected by temperature. Analysis of Beau-R N sgRNA levels showed that the quantity of this sgRNA was reduced during Beau-R infection, 6 to 24 hpi (*p* < 0.0005), at 41 °C when compared to synthesis at 37 °C (Figure 7b). This result implies a reduction in viral RNA synthesis at 41 °C, further indicating the role of the replicase gene for the restriction in growth of Beau-R at 41 °C. Interestingly, at the first time point for analysis, 4 hpi, the quantity of N sgmRNA was higher at 41 °C (*p* < 0.0001); this was also observed in M41-CK infection but the difference was not statistically significant. The titres of infectious progeny generated from both M41-CK (Figure 7c) and Beau-R (Figure 7d) infections were reduced from 10 hpi in cells incubated at 41 °C in comparison to 37 °C (*p* < 0.05). The reduction in Beau-R titres, however, were much more apparent, and alongside the reduction in N sgRNA levels (Figure 7b), suggests that the reduction in viral titre is likely the result of decreased viral RNA synthesis. The indication of comparable RNA synthesis for M41-CK at both temperatures (Figure 7a) does not offer a clear explanation to the moderate drop in viral titre at 41 °C, indicating the role of another factor. Investigation into N protein production (Figure 8) also does not explain the moderate drop in viral titre. During Beau-R infection at 41 °C, at 8, 11 and 24 hpi there was markedly less N protein detected in comparison to 37 °C, which is likely the consequence of reduced N gene sgmRNA production (Figure 7b). During M41-CK infection there was little difference in the levels of N protein detected, again indicating that another factor is contributing to the decrease in viral titre. In summary, the analysis of RNA levels and protein expression with regards to the N gene, suggests that for Beau-R, RNA synthesis is negatively affected by incubation at 41 °C, contributing to reduced protein production and a decrease in the quantity of infectious progeny virions. For M41-CK, RNA levels and protein expression were largely unaffected by temperature, however there was a moderate decrease in viral titre after 10 hpi at 41 °C.

### 3.9. Beau-R Replication at 41 °C Can Be Rescued by M41-CK

**Figure 9:**
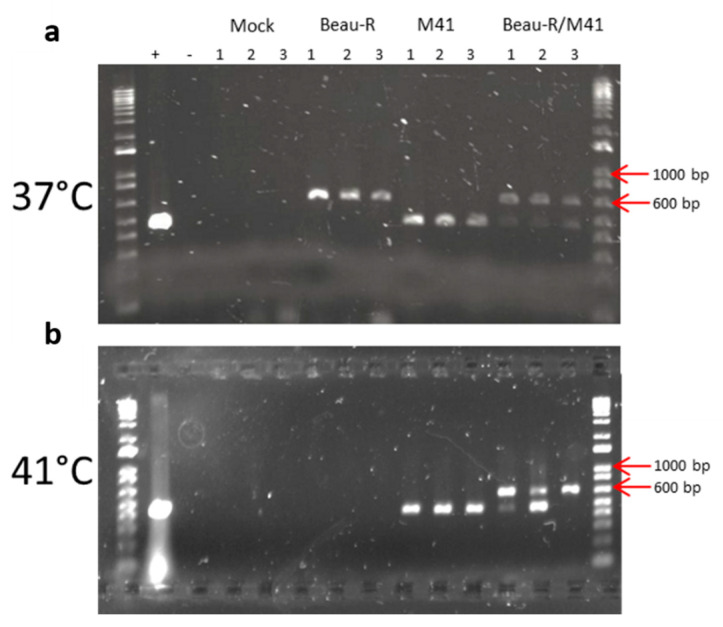
Beau-R replication at 41 °C can be rescued by M41-CK. Primary CK cells seeded in 6 well plates were inoculated with 10^5^ PFU (MOI ~0.01) of either M41-CK or Beau-R, or co-infected with 10^5^ PFU Beau-R and 10^5^ PFU M41-CK. BES media was used for mock infection. Cells were incubated at (**a**) 37 °C or (**b**) 41 °C, until extensive CPE was observed in the 37 °C wells. Progeny virus present in the supernatants were passaged a further four times in CK cells at the same temperature as the first passage. RNA was extracted from the final supernatants and assessed for the presence of M41-CK and Beau-R genomic material by (RT-PCR) analysis using primers targeting the 3′ UTR. RT-PCR products were separated by agarose gel electrophoresis alongside a 1 KB+ DNA ladder (Invitrogen). The red arrow highlights the marker bands relating to 1000 and 600 bp. RT-PCR products relating to Beau-R are ~650bp and M41-CK ~450 bp.

Our results indicate that a component of the IBV replicase protein, playing some role in RNA synthesis, was responsible for the restricted growth of Beau-R at 41 °C. To determine whether this was a dominant feature, we decided to assess whether Beau-R replication could restrict replication of M41-CK, or alternatively whether Beau-R replication could be rescued by M41-CK at 41 °C. CK cells were co-infected with Beau-R and M41-CK and incubated at either 37 °C or 41 °C. Progeny viruses in the resultant supernatants were subsequently passaged a further four times in CK cells at the temperature at which the first stage occurred. The presence of Beau-R and M41-CK genomic material was detected using an RT-PCR assay targeting the 3′ UTRs [23] (Figure 9). RT-PCR products derived from Beau-R and M41-CK RNAs, 667 and ~483 nucleotides, respectively, were detected either in CK cells infected with a single virus, or in cells co-infected with both viruses at 37 °C (Figure 9a). As expected, M41-CK RNA was detected in CK cells incubated at 41 °C, whereas Beau-R RNA was not detected. Both Beau-R and M41-CK derived RNA was detected in CK cells co-infected with both viruses at 41 °C (Figure 9b). These results show Beau-R RNA cannot be detected in CK cells passaged at 41 °C but can be detected in CK cells co-infected with M41-CK, demonstrating that the M41-CK replicase can rescue the replication of Beau-R genomic RNA at the non-permissive temperature. This indicates that during coinfection at the non-permissive temperature the M41-CK replicase is the dominant activity and the Beau-R replicase does not have a negative effect on the replication of M41-CK. Overall, the results of the co-infection experiments indicates that Beau-R and M41-CK can infect the same cell at the same time, and that incubation at 41 °C does not result in permanent or non-recoverable damage to the Beau-R RNA, providing further evidence that the Beau-R replicase is responsible for the observed restriction in replication of Beau-R RNA at 41 °C and that the effect is not due to an impediment with the Beau-R RNA.

## 4. Discussion

For birds, as well as mammals, the continuous movement of air through the respiratory tract results in a temperature gradient in which the upper respiratory tract, including the nasal cavities, mouth and throat, is cooler than the trachea and lungs that constitute the lower respiratory tract [43]. Research on human pathogens, including influenza virus, rhinovirus and RSV, has indicated that the temperature of the upper respiratory is between 33–34 °C and the lower to be 37 °C, commonly denoted as permissive and non-permissive temperatures, respectively [30,31,32,50,51,52]. Unlike humans which have a core body temperature of 37 °C, chickens exhibit a core temperature of 41 °C [48], which means it is likely that the temperature of the upper respiratory tract is 37–38 °C, increasing to 41 °C in the lower respiratory tract. The effect of this localised temperature change on the replication of several respiratory pathogens is well documented and has been linked to viral dissemination through the host, viral pathogenicity and interspecies transmission [30,31,32,52,53,54,55].

The data presented in this study demonstrates that the attenuated IBV strain, Beau-R, exhibits a temperature sensitive replication phenotype and is unable to replicate productively at 41 °C. This temperature sensitivity may account for the attenuated phenotype observed during in vivo infection (Figure 1), as it will inevitably contribute to the inability of Beau-R to disseminate from the point of inoculation to the lower respiratory tract. Infectious Beau-R was only detected in harvested beak tissue (Table 1) and was not detected in the trachea or the eyelid, unlike M41-CK that was shown to replicate in these sites and was shown to replicate successfully at 41 °C. Unlike Beau-R, the replication of other attenuated IBV isolates, including the vaccine H120, were found not to be temperature sensitive (Figure 3), indicating that temperature sensitivity may not be a universal cause of attenuation or loss of virulence. More importantly, however, another pathogenic isolate of IBV investigated that can also replicate in primary CK cells, D388(QX) was, in addition to M41-CK, able to replicate at 41 °C (Figure 4). This may suggest that the ability to replicate at higher temperature may be a shared characteristic of pathogenic isolates of IBV, and in particular nephropathogenic strains that have to disseminate deep into the body of the bird, to infect the kidneys, oviducts and enteric tract. This latter point is of particular interest as the replication kinetics of D388(QX) were found to be comparable at 37 °C and 41 °C (Figure 6) whereas M41-CK, a classical respiratory strain, was able to replicate at 41 °C but did experience a moderate reduction in viral titre at the higher temperature. This may, in part, explain why M41 is less often detected in the kidney during in vivo infection. To conclusively state that the ability to replicate at higher temperatures is a shared characteristic of pathogenic isolates further investigation of more IBV strains, which is difficult as many IBV pathogenic field strains cannot be propagated in vitro, will be required.

Temperature sensitivity is not a novel topic in the field of virology and has been widely studied for RNA viruses since the mid-1900s [43,56,57]. The analysis of temperature sensitive mutants has been used to identify genes involved in viral replication and pathogenesis as well as aiding in the generation of vaccine viruses [29,46,47,55,58,59,60,61,62,63]. Whilst temperature sensitivity has not been a popular research subject for IBV, [64,65,66], several temperature sensitive mutants of the *Betacoronavirus* mouse hepatitis virus (MHV) have been generated [55,62,67]. These mutants all carry mutations within the replicase gene, supporting the data presented in this study that the Beau-R replicase gene is a determinant of temperature sensitivity (Figure 6). These mutants also have defects in viral RNA synthesis [62]. The analysis of N protein sgRNA levels indicates that Beau-R also experiences defects in RNA synthesis at higher temperatures (Figure 7) but further research is required to elucidate both the extent of these defects and the mechanism behind them. The cause of the moderate reduction in M41-CK titres observed at 41 °C also requires further investigation as the data described here indicates that this is not the result of disrupted RNA synthesis (Figure 7). It is possible that protein–protein interactions, processing of the polyprotein, proteolytic cleavage or protein stability and/or activity are affected [55,67,68,69] or even that the virus-host response has been altered [31,55,67]. Interestingly a recent publication examining the effect of temperature on the replication on both SARS-CoV and SARS-CoV-2 has highlighted a temperature dependent type I interferon mediated antiviral response [70].

Regardless of the cause of Beau-R temperature sensitivity, the identification of a temperature sensitive replication phenotype, alongside restricted replication in vivo, may have implications for the further development of Beau-R as a vaccine vector. It is well established that the Beaudette strain replicates poorly in vivo, with the molecular clone Beau-R behaving comparably [15]. Additionally, vaccination of birds with Beau-R does not induce a protective immune response against M41-CK challenge [15]. Although vaccination with Beau-R expressing heterologous S genes can induce a partial protective immune response against homologous challenge, it does not meet the standards set by the European Pharmacopoeia, with one hypothesis reasoning that this is the result of poor levels of in vivo replication [15,16,20].

Beau-R is, however, a powerful molecular tool for coronavirus research. The temperature sensitive mutants identified for MHV have proved extremely useful in elucidating the specific roles of individual replicase proteins (nsps) as well as protein-protein and protein-RNA interactions during the coronavirus replication cycle [55,62,67,71]. Furthermore, temperature sensitive mutants have identified pathogenic determinants within the MHV genome and have also been demonstrated to protect mice against lethal challenge [55,67]. Temperature sensitivity has been used in development of vaccines against RSV [46] and influenza virus [32,61]. It is therefore possible, with further research into temperature sensitive lesions within the Beau-R replicase gene, that temperature sensitivity could be used to achieve attenuation of other IBV strains for future vaccine development, as well as a research tool to investigate the functions of the IBV replicase proteins.

## 5. Conclusions

The *Gammacoronavirus* IBV is a highly contagious economically important respiratory pathogen of domestic fowl. Current live attenuated vaccines are generated through serial passage of a virulent isolate through embryonated hens’ eggs; the molecular mechanism is unknown. The research described here identifies that the replication of the attenuated IBV strain Beau-R is temperature sensitive and is highly restricted in chickens to the soft tissue within the beak. The temperature sensitive replication phenotype of Beau-R was found to be associated with the replicase gene and with defects in sub-genomic mRNA synthesis. These findings highlight the possibility of utilising temperature sensitivity as a method for rational attenuation and vaccine development as seen with pathogens such as influenza virus.

## Figures and Tables

**Table 1:** The number of birds positive for virus isolation in each group.

Group	Dpi	Beak	Eyelid	Trachea
Mock	1	0/6	0/6	0/6
Beau-R	1	6/6	0/6	0/6
M41-CK	1	6/6	4/6	0/6
Mock	4	0/6	0/6	0/6
Beau-R	4	2/6	0/6	0/6
M41-CK	4	6/6	6/6	5/6
Mock	6	0/6	0/6	0/6
Beau-R	6	0/6	0/6	1/6
M41-CK	6	0/6	6/6	5/6
Mock	7	0/6	0/6	0/6
Beau-R	7	2/4	1/4	0/4
M41-CK	7	2/4 *	5/6	2/5 *

Notes: * Samples from the trachea of one bird and beak tissue from two birds were damaged during processing and therefore were unable to be included in the dataset. Beak refers to all soft tissue within the beak cavity, including nasal turbinates and nasal associated lymphoid tissue (NALT).
